# Supplementary material for: Functional comparison of distinct Brachyury+ states in a renal differentiation assay
Source: Biol Open. 2018 Apr 17;7(5):bio031799. doi: 10.1242/bio.031799 (PMC5992531; doi:10.1242/bio.031799)
Supplement: Supplementary information [file biolopen-7-031799-s1.pdf]

Supplementary Figures

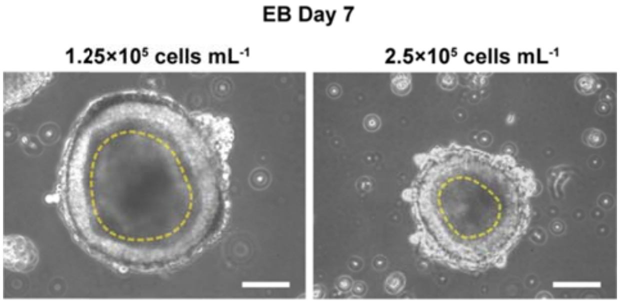

Fig. S1. Representative phase contrast photomicrographs of cavities within EBs derived from *Bra-GFP/Rosa26-E2C* mESCs at different seeding densities cultured for 7 days. Cells were seeded at densities of 2.5×10^5 and 1.25×10^5 cells mL^{-1}. Dashed lines show debris-filled cavities, consistent with the development of the proamniotic-like cavities as we have previously shown (see Fig. 3 in Murray and Edgar, 2000). Data were collected from three biological replicates. Scale bars: 100 µm.

Biology Open • Supplementary information

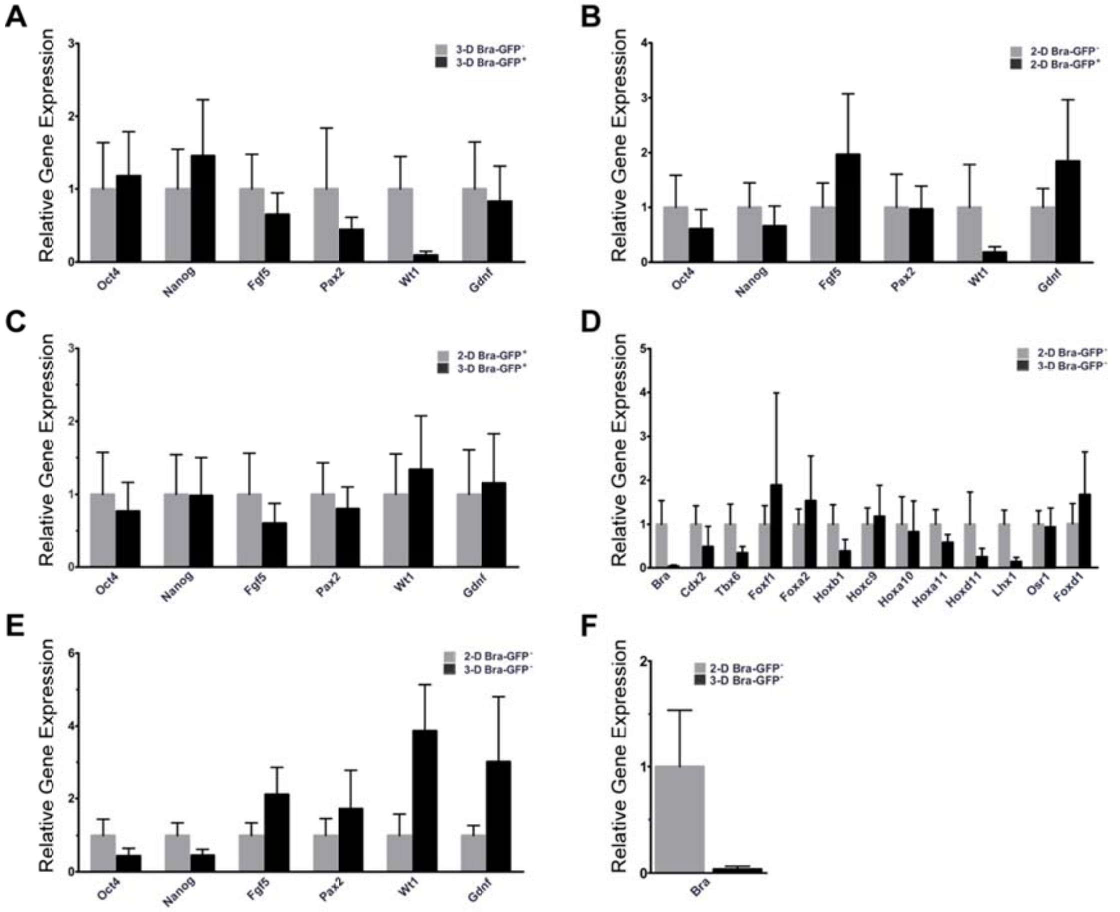

Fig. S2. qRT-PCR analysis of stemness and lineage markers expressed by the mesodermal and non-mesodermal populations from *Bra-GFP/Rosa26-E2C* mESCs cultured in 3-D and 2-D systems. (A) Relative expression levels of genes were compared between *Bra*-GFP$^+$ and *Bra*-GFP$^-$ populations isolated from the 3-D system (n=2 biological replicates), presented as mean±s.e.m. Data were not statistically assessed on significance due to there being two biological replicates however they gave an indication of the difference between *Bra*-GFP$^+$ and *Bra*-GFP$^-$ populations. (B) Relative expression levels of genes were compared between *Bra*-GFP$^+$ and *Bra*-GFP$^-$ populations isolated from the 2-D system (n=2 biological replicates), presented as mean±s.e.m. Data were not statistically assessed on significance due to there being two biological replicates however they gave an indication of the difference between *Bra*-GFP$^+$ and *Bra*-GFP$^-$ populations. (C) Relative gene expression levels genes were compared between *Bra*-GFP$^+$ populations isolated from 3-D system (n=3 biological replicates) and 2-D systems (n=3 biological replicates), presented as mean±s.e.m. P<0.05 was considered as statistically significant (t-test). No significant difference was found between the two systems. (D, F) Relative expression levels of mesoderm and early kidney development genes were

compared between *Bra*-GFP^{-} populations isolated from the 3-D and 2-D systems (n=2 biological replicates), presented as mean±s.e.m. Data were not statistically assessed on significance due to there being two biological replicates however they gave an indication of the difference between the two systems. Relative expression level of *Bra* is shown in (F). (E) Relative gene expression levels of stemness and key lineage genes were compared between *Bra*-GFP^{-} populations isolated from the 3-D and 2-D systems (n=2 biological replicates), presented as mean±s.e.m. Data were not statistically assessed on significance due to there being two biological replicates however they gave an indication of the difference between the two systems.

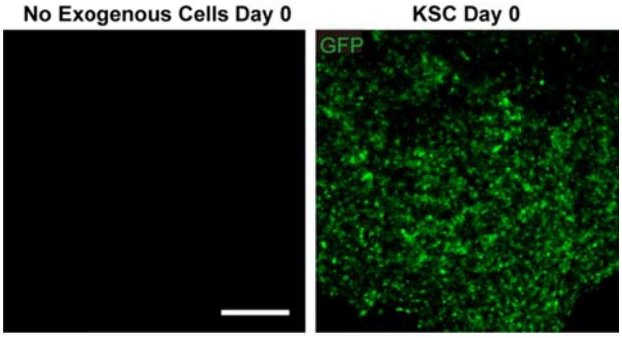

Fig. S3. Confocal photomicrographs of the re-aggregated E13.5 mouse embryonic kidney rudiments at day 0 of *ex vivo* culture containing no exogenous cells or GFP-KSCs. GFP-KSCs (positive controls) showed even distribution represented by GFP (green) in the rudiments at the beginning of the culture. Data were collected from three biological replicates. Scale bar: 200 µm.

Biology Open • Supplementary information

Supplementary Tables

Table S1 List of key genes investigated by qRT-PCR in this study

Genes	Expression Regions	References	Genes	Expression Regions	References
Bra	PS, TB, notocord	a, b	*Foxa2*	Anterior PS	m
Tbx6	PS, PM, TB	a-c	*Foxd1*	MM stroma	n
Cdx2	PS	d-f	*Foxf1*	LPM	o
Lhx1	LPM, IM	g	*Hoxa10*	PM, MM	e, p, q
Osr1	LPM, IM, MM	e, g	*Hoxa11*	PM, MM	e, p, q
Pax2	IM, ND, MM	g, h	*Hoxb1*	Posterior PS	m, r
Wt1	IM, MM	i	*Hoxc9*	Posterior PM	s
Gdnf	MM	j-l	*Hoxd11*	PM, MM	p, q

Notes: PS, primitive streak; PM, paraxial mesoderm; LPM, lateral plate mesoderm; IM, intermediate mesoderm; ND, nephric duct; MM, metanephric mesenchyme; TB, tailbud.

References: a, Papaioannou, 2014; b, Herrmann *et al*., 1990; c, Chapman *et al*., 2003; d, Arnold and Robertson, 2009; e, Taguchi *et al*., 2014; f, Savory, *et al*., 2009; g, Dressler, 2009; h, James and Schultheiss, 2005; i, Little, 2015; j, Lin *et al*., 1993; k, Sanchez *et al*., 1996; l, Basson *et al*., 2006; m, Gadue *et al*., 2006; n, Mugford *et al*., 2008; o, Mahlapuu *et al*., 2001; p, Carapuço *et al*., 2005; q, Yallowitz *et al*., 2011; r, Kmita *et al*., 2000; s, Erselius *et al*., 1990.

Biology Open • Supplementary information

Table S2 List of qRT-PCR primers*

Genes	Forward Sequences	Reverse Sequences	Amplicons Size (bp)	References
Bra	CATCGGAACAGCTCTCCAACCTAT	GTGGGCTGGCGTTATGACTCA	136	RTPrimerDB
β-actin	GTACCCAGGCATTGCTGACA	CTGGAAGGTGGACAGTGAGG	145	In-house
Gapdh	CATCTTCCAGGAGCGAGACC	GAAGGGGCGGAGATGATGAC	150	In-house
Fgf5	AAGTCAATGGCTCCCACGAA	TCCTCGTATTCCTACAATCCCCT	88	In-house
Foxd1	CAAGAATCCGCTGGTGAAGCC	ACAGGTTGTGACGGATGCTG	88	In-house
Foxf1	CCAAAACAGTCACAACGGGC	TCACACACGGCTTGATGTCT	191	In-house
Gdnf	CGCTGACCAGTGACTCCAAT	AAACGCACCCCCGATTTTTG	222	In-house
Nanog	AAGCAGAAGATGCGGACTGT	GTGCTGAGCCCTTCTGAATC	232	In-house
Oct4	TGGAGACTTTGCAGCCTGAG	CTTCAGCAGCTTGGCAAACTG	188	In-house
Osr1	GCCCCCAAAAAGGAGAGAGT	AGCCACAGCTCATCCTTTACC	161	In-house
Pax2	TCCAGGCATCAGAGCACATC	GGCCGATGCAGATAGACTGG	104	In-house
Wt1	AATGCGCCCTACCTGCCCA	CCGTCGAAAGTGACCGTGCTGTAT	116	In-house
Cdx2	QT00116739		114	Qiagen
Tbx6	QT00098861		80,80,157	Qiagen
Lhx1	QT01660792		87	Qiagen
Foxa2	QT00242809		115	Qiagen
Hoxa10	QT00240212		61	Qiagen
Hoxa11	QT00250404		97	Qiagen
Hoxb1	QT00493906		128	Qiagen
Hoxc9	QT00113218		138	Qiagen
Hoxd11	QT00267337		97	Qiagen

* Annealing temperature (Ta) is 60°C for all primers.

Supplementary Movies

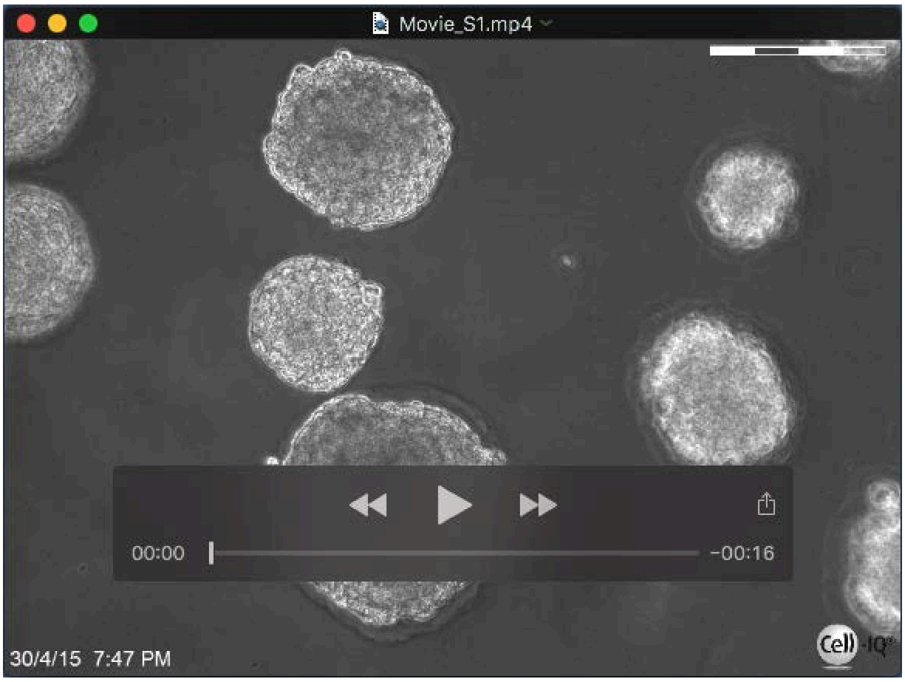

Movie S1: Representative Cell-IQ real-time imaging of day 3−8 EBs formed from *Bra-GFP/Rosa26-E2C* mESCs. EBs were seeded at the density of 1.25×10^5 cells mL^{-1}. Phase contrast images were acquired on an hourly basis. From around day 6, in some EBs, it appears that some cells started to migrate out, suggesting that they might be undergoing a gastrulation-like process (attached as a separate file).

Biology Open • Supplementary information

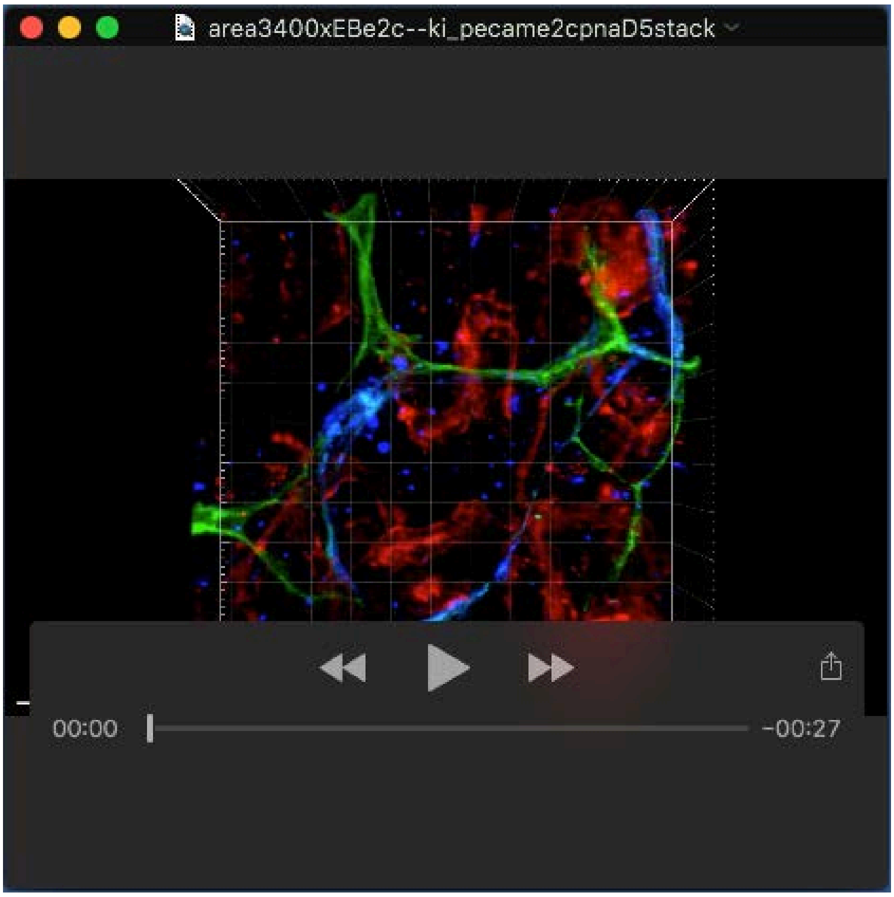

Movie S2: Representative 360-degree horizontal 3-D construction of confocal photomicrographs showing spatial distribution of PECAM-expressing 3-D system-derived E2C^{+} *Bra*-GFP^{+} cells within mouse embryonic kidney rudiments. Rudiments were cultured *ex vivo* for 5 days. Immunostaining for E2C (blue) and PECAM-1 (green) was performed to identify mesodermal and endothelial-like cells, respectively (attached as a separate file).

Biology Open • Supplementary information

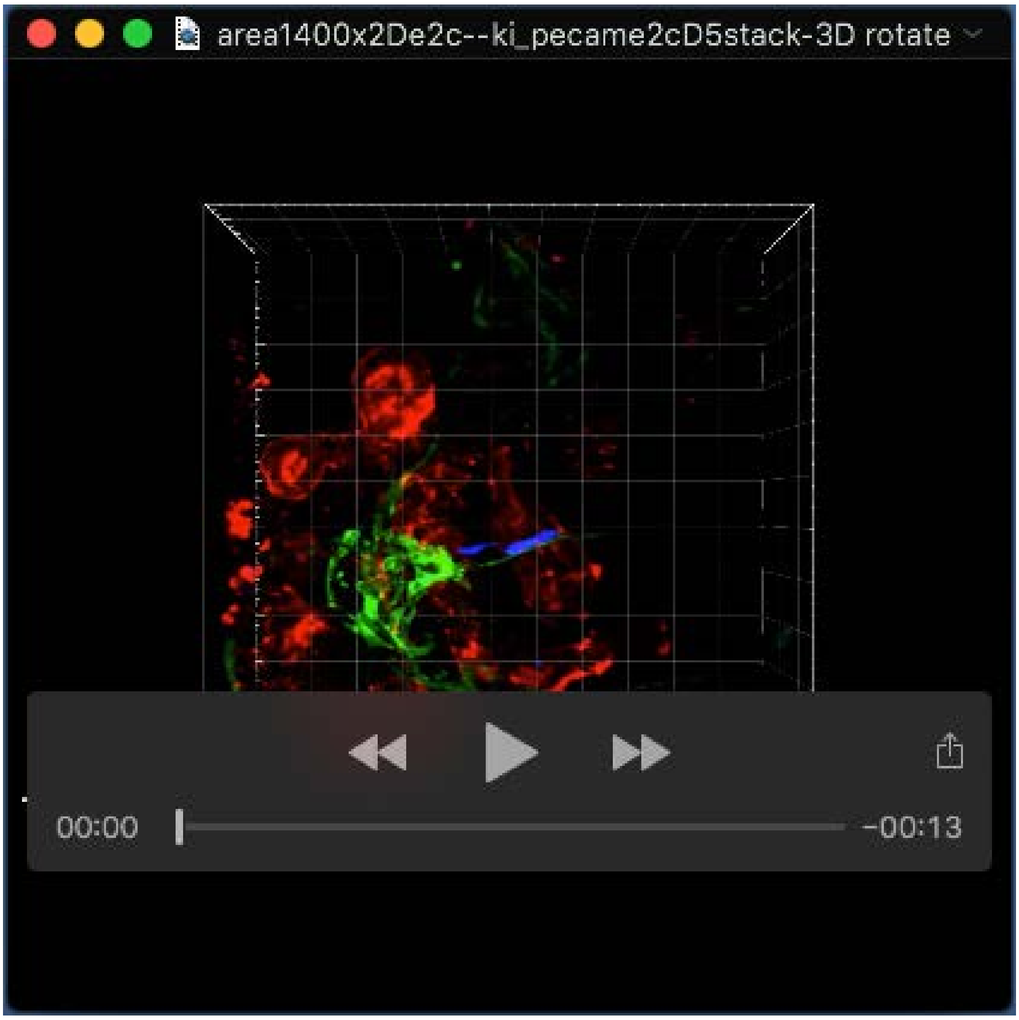

Movie S3: Representative 360-degree horizontal 3-D construction of confocal photomicrographs showing spatial distribution of PECAM-expressing 2-D system-derived E2C$^+$ *Bra*-GFP$^+$ cells within mouse embryonic kidney rudiments. Rudiments were cultured *ex vivo* for 5 days. Immunostaining for E2C (blue) and PECAM-1 (green) was performed to identify mesodermal and endothelial-like cells, respectively (attached as a separate file).

Biology Open • Supplementary information
